# Fabrication of Cellulose Derivatives-Based Highly Porous Floating Tablets for Gastroretentive Drug Delivery via Sugar Templating Method

**DOI:** 10.3390/polym17040485

**Published:** 2025-02-12

**Authors:** Pattaraporn Panraksa, Tanpong Chaiwarit, Baramee Chanabodeechalermrung, Patnarin Worajittiphon, Pensak Jantrawut

**Affiliations:** 1 Department of Pharmaceutical Sciences, Faculty of Pharmacy, Chiang Mai University, Chiang Mai 50200, Thailand; pattaraporn.pan@cmu.ac.th (P.P.); tanpong.ch@cmu.ac.th (T.C.); barameechana@gmail.com (B.C.); 2 Department of Chemistry, Faculty of Science, Chiang Mai University, Chiang Mai 50200, Thailand; patnarin.w@cmu.ac.th

**Keywords:** floating drug delivery, ethyl cellulose, hydroxypropyl methylcellulose, sugar templating, microstructural analysis, cellulose-based formulations, controlled drug release

## Abstract

This work presents an innovative application of the sugar templating method to fabricate highly porous floating tablets based on cellulose derivatives for gastroretentive drug delivery systems (GRDDS). Ethyl cellulose (EC) and hydroxypropyl methylcellulose (HPMC) were utilized to develop formulations that optimize porosity, buoyancy, and drug release. Among the tested formulations, E_10_H_5_/CPM, consisting of 10% *w*/*w* EC and 5% *w*/*w* HPMC loaded with chlorpheniramine maleate (CPM), exhibited the most favorable properties, including high porosity (94.4%), uniform pore distribution, immediate buoyancy, and over 24 h of floating time. E_10_H_5_/CPM tablets demonstrated superior drug release performance compared to an EC-only formulation (E_10_/CPM), attributed to the presence of HPMC, which facilitated improved hydration and diffusion. The *in vitro* release study showed that E_10_H_5_/CPM achieved a cumulative release of 79.01% over 72 h, following a Fickian diffusion mechanism. However, a limitation was noted in drug loading, with E_10_H_5_/CPM incorporating 6.40 mg of CPM, compared to 8.72 mg in E_10_/CPM. Future work should focus on enhancing drug load and further optimizing polymer composition to improve the release profile. Overall, this study underscores the potential of sugar templating in developing cost-effective, scalable floating tablet formulations for improved gastric retention and localized drug delivery.

## 1. Introduction

Gastroretentive drug delivery systems (GRDDS) have emerged as innovative drug delivery platforms designed to address the limitations of conventional oral drug delivery, such as poor bioavailability, fluctuating plasma drug levels, and inadequate drug release at the desired absorption site [1,2,3]. By extending gastric retention time, GRDDS offer enhanced therapeutic outcomes, particularly for drugs with a narrow absorption window (e.g., levodopa, furosemide, riboflavin) [4], those unstable in alkaline intestinal environments (e.g., verapamil, propranolol, diazepam) [5,6], or those requiring localized gastric delivery (e.g., ranitidine, or antibiotics for *Helicobacter pylori* eradication) [7,8]. Over the years, various types of GRDDS have been developed, including ion-exchange resin systems, magnetic systems, super-porous hydrogel systems, raft-forming systems, bioadhesive/mucoadhesive systems, expandable systems, high-density systems, and low-density/floating systems [9]. Among these approaches, floating drug delivery systems (FDDS) stand out as a particularly promising option due to their unique combination of simplicity, non-invasive nature, effectiveness, and minimal physiological interference. FDDS achieve buoyancy by reducing the dosage form density, thereby maintaining prolonged gastric residence without complex structural modifications or external devices. In contrast, other GRDDS approaches, such as high-density systems, face manufacturing challenges due to the need for increased dosage form size to achieve high drug load and desired densities [10], while magnetic systems generally rely on external devices that can hinder patient compliance and lead to practical challenges [11]. Similarly, mucoadhesive systems often face limitations from mucus turnover, variability in mucus composition, and the risk of unintended esophageal adhesion [12,13]. These limitations highlight the need for practical and patient-friendly FDDS with consistent performance characteristics.

To date, the manufacturing of FDDS has leveraged diverse fabrication techniques. For instance, Oh et al. employed the sublimation method and camphor to create highly porous metformin tablets with extended buoyancy. However, they encountered challenges in maintaining tablet strength with increased camphor content [14]. Similarly, Simons et al. utilized hot-melt extrusion (HME) to fabricate metformin FDDS in hollow tube forms, achieving immediate buoyancy dependent on the tube’s outer-to-inner diameter ratio [15]. More recently, additive manufacturing techniques, such as 3D and 4D printing, have been explored to design customizable FDDS with precise specifications [16,17,18]. While these methods offer advanced control, they involve significant costs, longer production times, and specialized expertise, making them less accessible for small-scale healthcare providers and pharmacists.

To address these challenges, we introduced the sugar templating method as a novel, accessible approach for fabricating highly porous floating tablets. This method involves using sugar or similar materials (e.g., citric acid monohydrate and sodium chloride) as a sacrificial template to create porous structures [19,20,21]. After the polymer matrix solidifies, the sugar is selectively removed through dissolution in water or an appropriate solvent, leaving behind a porous structure with interconnected pores mimicking the granular morphology of the template. This sugar-leaching process is critical to the technique, as it involves the careful removal of the sacrificial template without compromising the integrity of the surrounding polymer matrix, which retains its shape and structure. This simple, cost-effective, and environmentally friendly process has been widely employed in materials science. For example, González-Rivera et al. successfully fabricated highly porous polydimethylsiloxane (PDMS) foams using sugar templating, achieving interconnected 3D macrostructures with tunable pore sizes directly reflecting the sugar granule dimensions [22]. Similarly, in our prior work, we applied the sugar templating method to develop ethyl cellulose (EC) sponges, which demonstrated high porosity and well-connected pore networks suitable for various applications [23]. These studies underscore the versatility and potential of sugar templating in creating highly porous structures with tailored properties.

In this study, we extend the application of the sugar templating method to develop cellulose derivatives-based porous floating tablets for gastroretentive drug delivery, marking the first such application to our knowledge. Cellulose derivatives, such as EC and hydroxypropyl methylcellulose (HPMC), are widely utilized in pharmaceutical formulations due to their biocompatibility and favorable physicochemical properties. EC, a hydrophobic polymer, is water-insoluble but dissolves in organic solvents (e.g., alcohols, ketones, and aromatic hydrocarbons), forming robust matrices suitable for controlled drug release in gastric environments [24]. Its insolubility in water ensures structural integrity during the sugar-leaching process, allowing for the selective removal of the sacrificial sugar template without compromising the polymer matrix and making it an ideal candidate as the main polymer for sugar-templating processes. HPMC, on the other hand, is a hydrophilic cellulose ether that swells in water, creating gel-like structures that enable drug release via diffusion and erosion mechanisms [25]. The combination of these polymers allows fine-tuning of drug release profiles by adjusting the ratio of hydrophobic and hydrophilic components.

Overall, this study aimed to develop and evaluate cellulose derivative-based FDDS utilizing sugar templating to overcome the limitations of existing techniques. Highly porous floating tablets composed of EC and HPMC were fabricated, incorporating chlorpheniramine maleate (CPM) as a model drug. CPM, a widely used H_1_-receptor antagonist, is known for its synergistic effects with H_2_-receptor antagonists (e.g., famotidine) in reducing gastric inflammation and promoting ulcer healing [26]. By prolonging gastric retention, the developed floating tablets could enhance the stability and efficacy of CPM in localized gastric treatment while reducing the dosing frequency, thereby improving patient compliance and therapeutic outcomes. The formulations were also evaluated for their morphological properties, mechanical strength, *in vitro* floatability, and drug release behavior to demonstrate their potential in gastroretentive drug delivery applications.

## 2. Materials and Methods

### 2.1. Materials

Ethyl cellulose (EC) powder with an ethoxy content of 48.6% *w*/*w* was obtained from Sigma-Aldrich^®^ (St. Louis, MO, USA). Hydroxypropyl methylcellulose E5 (HPMC E5, AnyCoat^®^-C AN5, substitution type 2910, viscosity 5 mPa·s) was purchased from Lotte Fine Chemical Co., Ltd. (Ulsan, Republic of Korea). Caster sugar (very fine granulated sugar) was purchased from Thai Roong Ruang Industry Co., Ltd. (Bangkok, Thailand). Acetone (≥99.5% purity) and 1 N hydrochloric acid (HCl) were purchased from RCI Labscan, Ltd. (Bangkok, Thailand), while ethanol (95.0%) was obtained from the Liquor Distillery Organization Thailand (Chachoengsao, Thailand). Chlorpheniramine maleate (CPM), used as the model drug in this study, was supplied by S. Tong Chemicals Co., Ltd. (Nonthaburi, Thailand). All other chemicals and reagents were of analytical grade.

### 2.2. Preparation of Floating Tablets

#### 2.2.1. Design and Production of PDMS Tablet Molds

**Figure 1:**
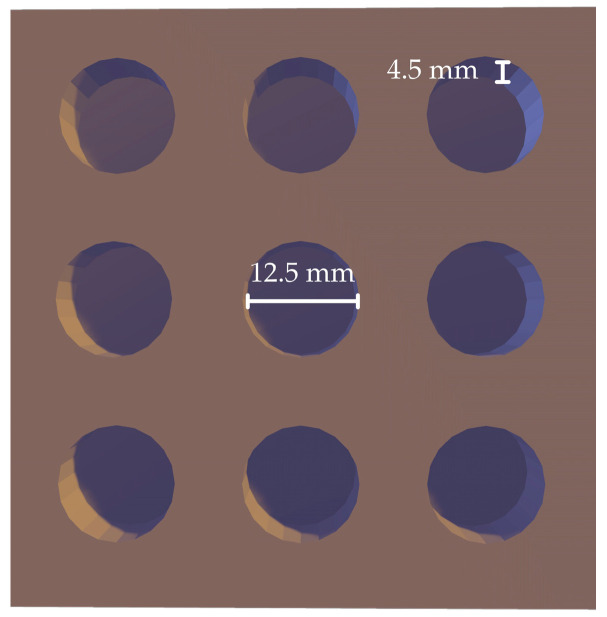
Schematic design of PDMS tablet molds.

The tablet molds were designed with specific dimensions of 12.5 mm in diameter and 4.5 mm in thickness (Figure 1). These dimensions were chosen to ensure easy handling, particularly for elderly patients, while maintaining a size suitable for comfortable swallowing. These molds were fabricated by the Biomedical Engineering Institute of Chiang Mai University using a vat polymerization technique. Polydimethylsiloxane (PDMS) was used as the mold material and mixed with a curing agent in a 10:1 weight ratio. The mixture was cured at 70 °C for at least 2 h to achieve a durable and flexible mold. Once cured, the molds were thoroughly cleaned and prepared for use.

#### 2.2.2. Preparation of Sugar Tablet Templates

**Figure 2:**
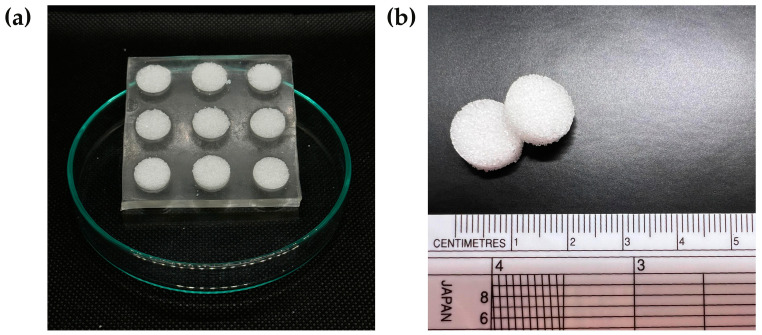
(**a**) Moist sugar pressed into molds; (**b**) sugar tablet templates after drying.

Sugar tablet templates were created by mixing caster sugar and distilled water in a ratio of 20:1 (*w*/*v*) to form a cohesive wet mass. The moistened sugar was then pressed into the PDMS molds to produce uniform sugar tablets with smooth surfaces (Figure 2a). Subsequently, the sugar-filled molds were oven-dried at 70 °C for 10 min. After drying, the sugar tablets (Figure 2b) were carefully removed from the molds to preserve their shape, ensuring they were suitable for subsequent fabrication steps.

#### 2.2.3. Fabrication of Floating Tablets Using the Sugar-Templating Method

The floating tablets in this study were fabricated using a sugar-templating approach. Initially, polymeric solutions were prepared by dispersing ethyl cellulose (EC) in acetone at varying concentrations, as detailed in Table 1. The solutions were stirred continuously at 200 rpm at room temperature (25 ± 2 °C) until homogeneous. In formulations containing hydroxypropyl methylcellulose (HPMC), EC was dispersed first, followed by the gradual addition of HPMC, with continuous stirring for 2 h or until the mixture was fully homogeneous. Next, the sugar tablet templates prepared in Section 2.2.2 (Figure 2b) were submerged in the EC or EC-HPMC mixtures and placed in a vacuum chamber for 10 min to remove air bubbles. The submerged templates were then left to stand for 2 h or until the polymeric mixtures had completely saturated the sugar tablets. Thereafter, the polymer-saturated sugar tablets were dried at 50 ± 2 °C to evaporate the acetone. The evaporation process was facilitated by the porous structure of the tablets. Once dried, the tablets were soaked in distilled water for 24 h to leach out the sugar and remove any residual acetone, as acetone is completely miscible with water. Subsequently, the resulting wet EC or EC/HPMC tablets were dried again in a hot air oven at 50 ± 2 °C to remove residual moisture. The water was periodically replaced throughout the process. These leaching and subsequent drying steps were repeated until the tablets reached a constant weight, ensuring the complete removal of residual water and acetone. Finally, all formulations were characterized to assess their morphology and identify those with optimal properties for drug loading and further analysis.

### 2.3. Morphological Analysis for Pre-Screening of Floating Tablet Formulations

All cellulose derivative-based floating tablet formulations underwent morphological analysis to pre-screen suitable candidates for drug loading. This evaluation aimed to ensure the completeness of the internal microstructure and identify formulations with porosity consistent with the sugar template. Tablets with large or irregular pores, indicative of incomplete polymer infiltration, were excluded from further testing due to inadequate structural integrity.

Initial screening was performed using a digital portable USB mini-microscope (Yao, Shenzhen, China) with a built-in LED light source and a high-definition CMOS sensor. The microscope was positioned at fixed distances of 2.0 and 5.0 cm from the samples to maintain consistent imaging conditions. Cross-sectional images were captured using Windows 10 Camera software version 2024.2408.1.0 (Microsoft Corporation, Redmond, WA, USA). For detailed structural evaluation, microstructural analysis of the cross-sections was conducted using a benchtop scanning electron microscope (SEM, JCM-7000 NeoScope™, JEOL Ltd., Tokyo, Japan). Before imaging, the tablets were halved to expose their cross-sections and mounted on aluminum stubs using double-sided conductive carbon tape (Nisshin EM Co., Ltd., Tokyo, Japan). The samples were gold-coated for 1 min using an automated sputter coater (JEOL Smart Coater, JEOL Ltd., Tokyo, Japan). SEM imaging was performed in a high vacuum with a secondary electron (SE) detector at an acceleration voltage of 15 kV and a magnification of ×27. Formulations exhibiting a homogeneous structure with suitable pore formation and uniform pore distribution were selected for subsequent drug-loading studies.

### 2.4. Fabrication of Drug-Loaded Floating Tablets

Chlorpheniramine maleate (CPM) was incorporated into selected floating tablet formulations by adding 4% *w*/*w* CPM to formulations E_10_ and E_10_H_5_, which will hereafter be referred to as E_10_/CPM and E_10_H_5_/CPM, respectively. The fabrication process for these drug-loaded floating tablets followed the same procedure as previously described in Section 2.2.2 and Section 2.2.3, with a slight modification to facilitate uniform drug dispersion. Specifically, CPM was first dissolved in acetone before the addition of EC to ensure homogeneous drug distribution within the polymeric matrix. The resulting drug-loaded tablets were subsequently characterized to evaluate drug incorporation, mechanical properties, floating performance, and drug release behavior.

### 2.5. Morphological Analysis of Drug-Loaded Floating Tablets

The morphology of the drug-loaded floating tablets was analyzed using the same methodology as described in Section 2.3 to assess the effect of CPM incorporation on tablet structure. Cross-sectional imaging was conducted to compare the internal microstructure of drug-loaded formulations (E_10_/CPM and E_10_H_5_/CPM) with their non-drug-loaded formulations, evaluating any changes in pore distribution and overall tablet morphology.

### 2.6. Physical Characterization of Drug-Loaded Floating Tablets

To ensure batch uniformity, the drug-loaded tablets were evaluated for weight variation, diameter, thickness, and density as follows:

#### 2.6.1. Weight Variation

Twenty tablets per formulation (n = 20) were individually weighed using an analytical balance with a readability of 0.1 mg (LAB 124i, Adam Equipment Co., Ltd., Milton Keynes, UK). The mean weight and standard deviation (SD) were calculated.

#### 2.6.2. Diameter and Thickness

Tablet diameter and thickness were measured using an electronic digital caliper with an accuracy of ±0.01 mm (Becthai Bangkok Equipment & Chemical Co., Ltd., Nakhon Pathom, Thailand). Measurements were taken at three different points on each tablet, and the average values were recorded.

#### 2.6.3. Tablet Density

The density (D) of the floating tablets was calculated using the following equation (Equation (1)) [14]:

(1) $$D=W/\left[{\left(M/2\right)}^{2}\times \pi \times h\right]$$

where W is the tablet weight (g), M is the tablet diameter (cm), and h is the tablet thickness (cm).

### 2.7. Porosity Analysis via Micro-CT Scan

The porosity and internal microstructure of the drug-loaded floating tablets were analyzed using a high-resolution micro-computed tomography (micro-CT) or x-ray computerized tomography scanner (SkyScan 1173, Bruker Corporation, Billerica, MA, USA). Scanning was performed at a voltage of 40 kV, a current of 100 µA, and a pixel resolution of 12.82 µm. A full 360° rotation of each sample was completed with a step size of 0.3°. Projection images were reconstructed into cross-sectional slices using NRecon software (version 1.6.9.18, Bruker Corporation). 3D models were generated using Dragonfly software (version 2024.1, Comet Technologies Canada Inc., Montréal, QC, Canada) [27] to visualize the structure and porosity inside the structure. Porosity quantification was performed using CTAn software (version 1.10, Bruker Corporation), which calculated open porosity percentages. Thresholding was applied to differentiate solid and void regions within the tablet structure. Additionally, radiodensity measurements of the drug-loaded floating tablets were obtained using Dragonfly software to further characterize their physical properties.

### 2.8. Mechanical Strength Testing of Drug-Loaded Floating Tablets

The mechanical strength testing of the floating tablets was evaluated using a texture analyzer (TA.XTplus, Stable Micro Systems Ltd., Surrey, UK), following methods adapted from Thapa et al. [28] and Farias et al. [29], with modifications in instrumentation and testing conditions. The test was conducted at room temperature (25 ± 2 °C) using a 5 kg load cell and a 2 mm stainless steel cylindrical probe (P/2 probe). Before testing, the tablet diameter and thickness were measured using an electronic digital caliper. The texture analyzer operated in compression mode with a pre-test speed of 2.0 mm/s, a test speed of 1 mm/s until the probe penetrated 10 mm into the sponge-like structure of the tablet, and a post-test speed of 10 mm/s. The maximum force required for the probe to penetrate the tablet was recorded as the resistance force.

The radial tensile strength ($\sigma_{x}$) of the floating tablets was calculated using Equation (2) [28]:

(2) $$\sigma_{x}=\frac{2F}{\pi dt}$$

where $\sigma_{x}$ represents the tensile strength (MPa), F is the maximum resistance force (N), d is the tablet diameter (mm), and t is the tablet thickness (mm).

All measurements were performed in quintuplicate (n = 5), and results were reported as mean ± SD.

### 2.9. Determination of CPM Loading Content

The CPM content in the floating tablets was determined by dissolving three tablets from each formulation (E_10_/CPM and E_10_H_5_/CPM) individually in 10 mL of 95% *v*/*v* ethanol under magnetic stirring at 200 rpm and 25 ± 2 °C for 2 h. Then, the resulting solution was diluted 20-fold with 95% ethanol, filtered through a 0.45 µm nylon syringe filter (Labfil^®^, Zhejiang Alwsci Technologies Co., Ltd., Hangzhou, China) and analyzed via UV-VIS spectrophotometry (UV-2600i, Shimadzu Corporation Kyoto, Japan) at a wavelength of 262.0 nm. The CPM content was quantified using a standard calibration curve, which was constructed for CPM in water over a concentration range of 5–60 µg/mL. The calibration curve demonstrated linearity with a high correlation coefficient (R^2^ = 0.9992). The linear regression equation obtained was *y* = 0.0147*x* − 0.0368, where *y* represents absorbance and *x* corresponds to CPM concentration (µg/mL).

### 2.10. In Vitro Buoyancy Studies

The floating behavior of the CPM-loaded floating tablets (E_10_/CPM and E_10_H_5_/CPM) was evaluated using an *in vitro* buoyancy test, adapted from the method described by Nigusse et al. [30], with modifications to align with the USP monograph for CPM tablet analysis [31]. Each tablet was placed in a 100-mL beaker containing 100 mL of 0.01 N hydrochloric acid (HCl) as the dissolution medium. The medium was maintained at 37 ± 0.5 °C and stirred at a rotation speed of 50 rpm. The floating lag time (i.e., the time required for the tablet to rise to the medium surface) and the total floating time (i.e., the time the tablet remained buoyant on the medium surface) were visually recorded.

### 2.11. In Vitro CPM Release Studies and Drug Release Kinetics

The drug release characteristics of the floating tablets were investigated under the same conditions as the *in vitro* buoyancy test. The dissolution study was conducted in a 100-mL beaker containing 100 mL of 0.01 N HCl, maintained at 37 ± 0.5 °C, and stirred at 50 rpm using a magnetic stirrer. The experiment was conducted in triplicate, with 3 mL samples collected at predetermined time points (0.5, 1, 2, 3, 4, 5, 6, 7, 8, 12, 18, and 24 h) to monitor the drug release. After each sample withdrawal, an equal volume of fresh dissolution medium was added to maintain sink conditions. The collected samples were subsequently filtered using a 0.45 µm nylon syringe filter, diluted as necessary, and analyzed using a UV-VIS spectrophotometer (UV-2600i, Shimadzu Corporation, Kyoto, Japan) at λ_max_ of 264.0 nm. The amount of drug release at each time point was calculated based on a standard calibration curve derived from the absorbance-concentration relationship in 0.01 N HCl (*y* = 0.0211*x* − 0.0241, R^2^ = 0.9994, where *y* and *x* correspond to absorbance and CPM concentration (µg/mL), respectively). The results were graphically represented as cumulative drug release (%) over time (h).

To characterize the drug release mechanisms, the release data were fitted to various mathematical models, including zero-order, first-order, Higuchi, and Korsmeyer-Peppas (KP) models. The corresponding equations for each kinetic model are presented below (Equations (3)–(6)):

(3) $$\text{Zero-order}\;\mathrm{model}:\;Q_{t}=Q_{0}+k_{0}\cdot t$$

where $Q_{t}$ is the amount of drug released at time t, $Q_{0}$ is the initial drug amount, and $k_{0}$ is the zero-order rate constant.

(4) $$\text{First-order}\;\mathrm{model}:\;\ln Q_{t}=\ln Q_{0}-k_{1}\cdot t$$

where $Q_{0}$ and $Q_{t}$ represent the initial and released drug amount at time t, respectively, and $k_{1}$ is the first-order rate constant.

(5) $$\mathrm{Higuchi}\;\mathrm{model}:\;Q_{t}=k_{H}\cdot t^{1/2}$$

where $Q_{t}$ is the drug amount released at time t, and $k_{H}$ is the Higuchi diffusion constant.

(6) $$\mathrm{Korsmeyer}–\mathrm{Peppas}\;\mathrm{model}:\;\frac{Q_{t}}{Q_{\infty }}=k_{K}\cdot t^{n}$$

where $\frac{Q_{t}}{Q_{\infty }}$ represents the fraction of drug released at time t, $k_{K}$ is a constant related to the structural and geometric properties of the dosage form, and n is the release exponent.

### 2.12. Statistical Analysis

All data are presented as mean ± standard deviation (SD). Statistical analyses were performed using one-way analysis of variance (ANOVA) with SPSS^®^ Statistics software version 17.0 (IBM Corporation, Armonk, NY, USA). A *p*-value < 0.05 was considered statistically significant.

## 3. Results and Discussion

### 3.1. Morphological Analysis of Cellulose Derivative-Based Floating Tablets

**Figure 3:**
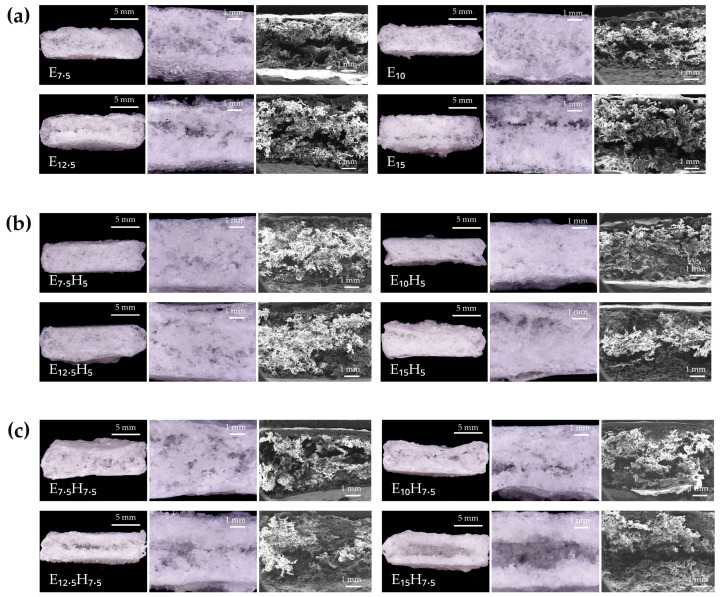
Cross-sectional images of cellulose derivative-based floating tablet formulations: (**a**) EC-only formulation, (**b**) EC with 5% *w*/*w* HPMC, and (**c**) EC with 7.5% *w*/*w* HPMC.

The morphological characteristics of cellulose derivative-based floating tablets were examined using both digital microscopy and SEM to evaluate their internal structure, with a focus on porosity, uniformity, and pore size distribution. Cross-sectional SEM images of the floating tablets (Figure 3) revealed notable differences in pore structures among the various formulations, primarily influenced by the concentrations of EC and HPMC in the polymeric mixtures. Despite these variations, all tablets exhibited a highly porous and sponge-like structure, which is crucial for maintaining buoyancy.

In formulations containing only EC (Figure 3a), pore structures varied significantly based on polymer concentration. At low EC concentrations (E_7.5_), the tablets exhibited large and irregular hollow regions, likely due to insufficient polymer content to fully form a stable matrix. Conversely, at very high EC concentrations (E_12.5_ and E_15_), the tablets displayed elongated hollow channels and irregularly shaped large pores, potentially resulting from the high viscosity of the polymeric solution. The high viscosity likely hindered the effective infiltration of polymeric solution into the sugar template, leading to incomplete pore formation. This observation is consistent with the findings by Prasanthi et al. [32], which found that highly viscous liquids tended to diffuse less effectively into porous structures, resulting in reduced infiltration efficiency compared to lower-viscosity liquids. Similarly, in this study, the elevated viscosity of the EC solutions hindered complete infiltration into the sugar template, thereby affecting the uniformity and quality of the pore network. Notably, E_10_ exhibited the smallest and most uniform pore distribution, indicating an optimal balance between solution viscosity and template infiltration. These findings highlight that both excessively low and excessively high EC concentrations resulted in suboptimal morphology.

The addition of HPMC to the formulations significantly influenced the internal morphology of the tablets. Formulations containing 5% *w*/*w* HPMC (Figure 3b) exhibited a more homogeneous pore distribution compared to EC-only formulations. Among these, E_10_H_5_ demonstrated a well-defined and uniform pore structure, suggesting that the synergistic effects of EC and HPMC enhanced the polymeric network. This enhancement can be attributed to the ability of EC and HPMC to form hydrogen bonds and hydrophobic interactions. EC, being relatively hydrophobic, interacted with the hydrophobic regions of HPMC, while hydroxyl groups in both polymers enabled hydrogen bonding [33]. These interactions likely contributed to the formation of a more cohesive and stable polymer matrix, thus improving pore uniformity and structural integrity. However, when the HPMC content increased to 7.5% (E_7.5_H_7.5_, E_10_H_7.5_, E_12.5_H_7.5_, and E_15_H_7.5_), the SEM images (Figure 3c) revealed larger hollow cavities and more irregular pores. These changes could be attributed to the increased viscosity of the EC-HPMC mixture, which reduced the efficiency of polymer infiltration into the sugar template. Additionally, the high viscosity may have exerted a physical force on the sugar particles during the soaking process, disrupting their arrangement and resulting in wider pore spacing. Similar disruptions in pore uniformity were also reported by Oh et al. in a study where templating systems were fabricated using highly viscous polymeric solutions [34]. Moreover, the excessive HPMC content likely increased the matrix’s hydrophilicity, which influenced pore formation during the sugar-leaching and drying processes. Since HPMC is highly hydrophilic, it may have absorbed and retained more water during the sugar-leaching step, causing the polymeric network to swell. This swelling could have disrupted the pore uniformity, leading to the formation of larger and more irregular pores during drying as the absorbed water evaporated [35]. Rapid moisture removal during drying may have also caused structural shrinkage or deformation [36], further contributing to the inconsistent porous morphology observed in formulations with 7.5% *w*/*w* HPMC. These findings underscore the importance of balancing HPMC content to maintain a desirable pore structure and ensure structural stability.

Based on these results, E_10_ and E_10_H_5_ were selected for drug loading and further study due to their well-defined and fully porous structures achieved through sugar templating, indicating their potential for optimized drug delivery performance.

### 3.2. Impact of CPM Incorporation on Tablet Morphology

**Figure 4:**
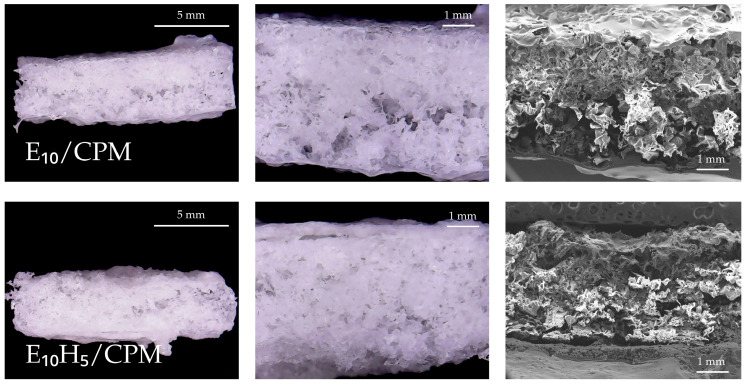
Cross-sectional images of drug-loaded floating tablets.

The incorporation of CPM into the selected floating tablet formulations (E_10_/CPM and E_10_H_5_/CPM) caused subtle yet significant changes in pore morphology. Cross-sectional SEM images of the drug-loaded formulations (Figure 4) revealed slightly reduced pore sizes compared to their non-drug-loaded formulations. The reduction likely occurred because CPM occupied pore spaces or altered the polymeric matrix during solvent evaporation. Specifically, as acetone evaporated during drying, CPM molecules may have precipitated within the polymeric network, creating additional solid material that partially filled or narrowed the pores. This result aligns with previous publications that demonstrated decreased porosity when additional compounds, such as graphene, were incorporated into polymeric matrices. Earlier work on PDMS foams reported a reduction in porosity with increasing graphene content, with a significant decrease observed at 30% graphene concentration. Similarly, our findings suggested that CPM incorporation led to a denser tablet structure due to reduced pore size [37]. In addition, macroscopic images of the floating tablets in Figure S1 confirmed that the overall size of the fabricated floating tablets was consistent with the sugar tablet template used during preparation. The uniform dimensions of the tablets, as shown in both images, suggested precise replication of the template mold, ensuring consistency across different batches. This observation further supported the structural integrity and reliability of the templating method used in this study.

### 3.3. Physical and Microstructural Characteristics of Drug-Loaded Floating Tablets

The physical and microstructural characteristics of drug-loaded floating tablets are critical parameters influencing the overall performance of a floating drug delivery system. In this study, formulations E_10_/CPM and E_10_H_5_/CPM were evaluated for their physical properties, including weight, diameter, thickness, density and porosity, with results summarized in Table 2.

Significant differences in tablet weight were observed between the formulations. E_10_/CPM had an average weight of 39.93 ± 0.25 mg, whereas E_10_H_5_/CPM was significantly heavier at 50.40 ± 3.69 mg (*p* < 0.05). The higher weight of E_10_H_5_/CPM can be attributed to the presence of 5% *w*/*w* HPMC, which increased the polymer content within the matrix. Furthermore, the observed variation in tablet weight between E_10_/CPM and E_10_H_5_/CPM suggested differences in polymer deposition on the tablet surface and infiltration ability into the internal structure of the sugar template. Since E_10_H_5_/CPM contained a blend of EC and HPMC, its polymeric solution had a higher viscosity compared to the EC-only formulation (E_10_/CPM). This increased viscosity likely resulted in a thicker polymer coating on the tablet surface while also allowing some degree of polymer penetration into the internal porous network of sugar template.

**Figure 5:**
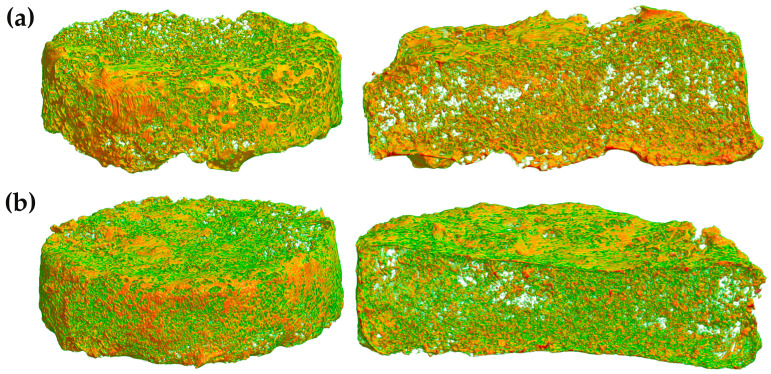
X-ray micro-CT images showing full and cross-sectional views of (**a**) E_10_/CPM and (**b**) E_10_H_5_/CPM floating tablets.

Micro-CT 3D imaging (Figure 5) further supports these findings, illustrating distinct differences in surface coating and internal polymer distribution between the two formulations. The E_10_H_5_/CPM tablets appeared to have a denser polymeric layer on the exterior while also showing evidence of polymer infiltration into the internal pores, which may have contributed to their higher density (0.095 ± 0.010 g/cm^3^) compared to E_10_/CPM (0.079 ± 0.003 g/cm^3^). In contrast, the E_10_/CPM tablets exhibited a less compact polymer network and a more open structure, explaining their lower density and reduced mass. The micro-CT images also indicate that the surface of E_10_/CPM tablets was less tightly packed compared to HPMC-containing formulations, further emphasizing the impact of polymer composition on tablet morphology. Additionally, open porosity measurements from micro-CT scans revealed significant differences between the two formulations, with E_10_H_5_/CPM exhibiting a porosity of 94.4% compared to 93.6% for E_10_/CPM. The higher open porosity of E_10_H_5_/CPM aligned with its denser polymer network, which likely facilitated better interconnectivity of pores during the sugar-leaching step.

In addition to porosity analysis, radiodensity measurements were conducted to further characterize the microstructure of the floating tablets. The obtained radiodensity values were −950.03 Hounsfield Units (HU) for E_10_/CPM and −943.76 HU for E_10_H_5_/CPM. These findings indicate that both formulations possessed highly porous structures, as negative radiodensity values (<0 HU) suggested air-filled voids within the matrix [38,39]. These results align with the observed density and micro-CT image data, where E_10_H_5_/CPM exhibited slightly denser structure compared to E_10_/CPM. This correlation further highlights the impact of polymer composition on the structural properties of the floating tablets.

Similarly, the tablet thickness followed a comparable trend, with E_10_H_5_/CPM (4.39 ± 0.14 mm) being slightly thicker than E_10_/CPM (4.30 ± 0.16 mm). Although the difference was not statistically significant, it suggested that the presence of HPMC contributed to a marginal increase in structural volume, likely due to the polymer’s ability to swell upon hydration during the sugar-leaching step. Compared to the original sugar template thickness (4.51 ± 0.12 mm), both formulations showed slight shrinkage, with E_10_/CPM exhibiting a more noticeable reduction due to its less dense polymer matrix.

In terms of tablet diameter, both formulations closely matched the diameter of the original sugar template (12.19 ± 0.02 mm), with E_10_/CPM having a diameter of 12.23 ± 0.04 mm and E_10_H_5_/CPM slightly larger at 12.43 ± 0.08 mm. This slight increase in diameter for E_10_H_5_/CPM was consistent with its higher polymer content, which contributed to a thicker coating and a marginally larger tablet size.

### 3.4. Mechanical Strength of Drug-Loaded Floating Tablets

The mechanical strength of the drug-loaded floating tablets was evaluated to assess their durability during handling and their potential to withstand mechanical stresses in the gastrointestinal environment. Radial tensile strength ($\sigma_{x}$) values for E_10_/CPM and E_10_H_5_/CPM formulations are presented in Table 3. The results showed that E_10_H_5_/CPM tablets exhibited significantly higher tensile strength (0.156 ± 0.004 MPa) compared to E_10_/CPM tablets (0.143 ± 0.045 MPa) (*p* < 0.05). This increase in tensile strength for E_10_H_5_/CPM may be attributed to the presence of HPMC, which enhanced the structural integrity of the polymer matrix by increasing polymer-polymer interactions and forming a more cohesive network. The reduced pore size, as discussed in Section 3.2, likely also contributed to the higher mechanical strength by decreasing void volume and increasing the effective solid content of the tablets. These findings align well with a previous study by Freag et al. [40], which developed buccal chitosan-based composite sponges. Their results demonstrated that the incorporation of HPMC into the formulations improved mechanical properties and increased the hardness of the chitosan sponges.

Generally, a tensile strength greater than 1.7 MPa is considered sufficient to ensure that a tablet is mechanically robust enough for manufacture and distribution [41,42]. However, in this study, the observed tensile strengths for both formulations were considerably lower than this threshold. This low tensile strength was likely due to their highly porous and sponge-like structure, which was specifically designed to enhance buoyancy and prolong gastric retention via floating. Such structural characteristics allow easy penetration by the texture analyzer probe, leading to lower tensile strength values. Similar trends have been observed in sponge-like wound dressings and other porous drug delivery systems, where increased porosity often resulted in reduced mechanical strength [43,44]. Our results are consistent with a previous study on sponge-like dressings, which exhibited comparable mechanical properties (approximately 0.07–0.20 MPa) [45]. This similarity suggests that such tensile strength values are characteristic and normal for sponge-like structures. To enhance mechanical robustness of the highly porous tablets, future studies could explore the incorporation of cross-linking agents such as boric acid to reinforce polymeric interactions [46]. Alternatively, blending EC with polymers such as methacrylic derivatives or Kollidon^®^ SR (a blend of polyvinyl acetate and PVP K30 in an 8:2 ratio) may help improve tensile strength [47,48]. While the low tensile strength observed in this study may limit the tablets’ ability to withstand high mechanical stress, it is unlikely to affect their practical use by patients if appropriate precautions are taken. To mitigate potential issues during handling and transportation, special packaging such as blister packs or individually compartmentalized containers should be employed. Such packaging can help protect the tablets from excessive mechanical forces, thereby ensuring their integrity until use.

### 3.5. Drug Loading Content

The CPM loading content in the floating tablets was determined to assess the efficiency of drug incorporation. As shown in Table 3, E_10_/CPM exhibited a CPM loading content of 8.72 ± 0.53 mg, whereas E_10_H_5_/CPM had a significantly lower loading of 6.40 ± 0.22 mg (*p* < 0.05). These drug amounts are sufficient to reduce the frequency of CPM administration, where the typical dose of CPM is 4 mg every 4–6 h. The lower drug content in E_10_H_5_/CPM may be attributed to the presence of HPMC, which potentially influenced drug retention within the polymer matrix. One possible explanation is that a portion of the CPM may have dissolved in water during the sugar-leaching step rather than being fully retained within the polymer network. Despite this, a substantial amount of CPM remained entrapped in the polymer matrix due to the hydrophobic nature of EC, which restricted drug diffusion into water. Additionally, solvent evaporation likely promoted CPM precipitation within the matrix [49,50], further minimizing drug loss. Since CPM is highly water-soluble (>100 mg/mL) [51], some degree of loss was anticipated during sugar-leaching. However, the narrow SD values in drug content analysis suggest that any drug loss was consistent across batches and did not introduce significant variability in drug content. These findings support the reproducibility of the manufacturing process and highlight the importance of optimizing polymer composition to maximize drug retention within floating tablets. To address potential drug loss, we optimized the leaching process by using the shortest possible time while ensuring complete removal of the sugar. This approach aimed to minimize unnecessary exposure to water, thereby reducing CPM dissolution and loss. Future studies may explore the incorporation of poorly water-soluble drugs, which could further reduce drug loss during sugar-leaching and improve drug retention within the matrix while maintaining the desired porous structure for floating and controlled drug release. This strategy could also contribute to extending dosing intervals, thereby enhancing therapeutic efficacy.

### 3.6. Floating Behavior of Drug-Loaded Floating Tablets

The floating behavior of the drug-loaded tablets was evaluated to determine their suitability for prolonged gastric retention. Both E_10_/CPM and E_10_H_5_/CPM demonstrated an immediate floating lag time of 0 min, indicating their ability to float upon contact with the dissolution medium. Additionally, both formulations exhibited a total floating duration exceeding 24 h, confirming their potential for extended gastric retention. The prolonged floating time can be attributed to the highly porous structure of the tablets, which effectively trapped air within the matrix, allowing them to remain buoyant. Furthermore, despite the slightly higher density of E_10_H_5_/CPM, its floating performance remained uncompromised, likely because its density was still lower than that of gastric fluid (1.004 g/cm^3^) [52,53]. This suggests that the polymeric composition maintained a balance between structural integrity and buoyancy. These findings indicate that the developed floating tablets possess excellent floating properties, making them suitable candidates for GRDDS.

### 3.7. In Vitro CPM Release Profiles and Release Kinetics

**Figure 6:**
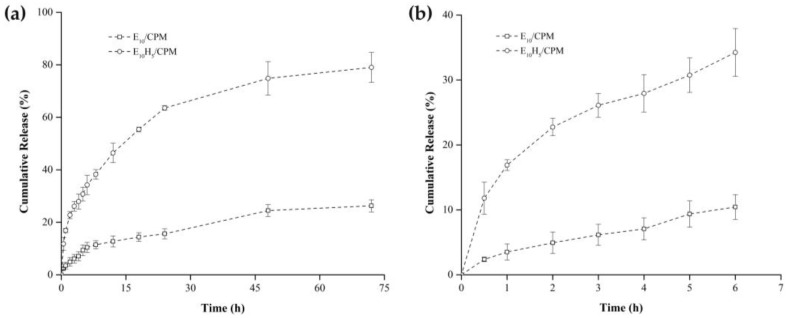
*In vitro* CPM release profiles of E_10_/CPM and E_10_H_5_/CPM floating tablets over 72 h (**a**) and the first 6 h (**b**).

The *in vitro* drug release profiles of the floating tablets (E_10_/CPM and E_10_H_5_/CPM) were investigated to evaluate their sustained-release behavior and assess the impact of polymer composition on drug release kinetics. The results revealed distinct differences between the two formulations in terms of cumulative drug release over time (Figure 6). The floating tablets containing only EC (E_10_/CPM) exhibited a markedly slower release rate compared to the HPMC-containing formulation (E_10_H_5_/CPM). At 72 h, E_10_/CPM released only 26.30 ± 2.31% of the drug, whereas E_10_H_5_/CPM achieved a substantially higher release of 79.01 ± 5.68%. These findings highlight the influence of HPMC on the release characteristics of the floating tablets. The limited drug release observed in the E_10_/CPM formulation can be attributed to the hydrophobic nature of EC [54], which serves as the main polymer in the matrix. EC creates a barrier that hinders the penetration of the dissolution medium into the tablet, thereby slowing the diffusion of CPM. Consequently, the drug release is prolonged but incomplete. In contrast, the addition of 5% *w*/*w* HPMC in the E_10_H_5_/CPM formulation significantly altered the release behavior. HPMC is a hydrophilic polymer that absorbs water and swells upon exposure to the dissolution medium, forming a gel-like layer on the tablet surface. This gel layer enhances matrix hydration and provides channels for the diffusion of CPM, leading to more efficient and sustained drug release [55]. The presence of HPMC thus counterbalances the hydrophobic effect of EC, facilitating improved drug release. These findings are consistent with the findings of Yang et. al. [54], who fabricated tablets via 3D printing using EC as the main polymer and investigated the influence of release modifiers (e.g., HPMC, sodium alginate (SA)) on drug release behavior. Their study demonstrated that without release modifiers, drug release through the matrix was limited (17.8% in 24 h). However, when HPMC and SA were added as release modifiers, the drug release within 24 h increased significantly to 83.0% and 80.3%, respectively. This underscores the critical role of hydrophilic additives in overcoming the limitations of hydrophobic matrices and enhancing drug release efficiency.

The release mechanisms of these formulations were further investigated by fitting the release data to various mathematical models, including zero-order, first-order, Higuchi, and Korsmeyer-Peppas models. The best-fitting model for each formulation was identified based on the highest regression coefficient (R^2^) values, as presented in Table 4. The results indicated that the release kinetics of E_10_/CPM were best described by the zero-order model (R^2^ = 0.9901) and the Korsmeyer-Peppas model (R^2^ = 0.9900, n = 0.64). The zero-order model, characterized by a constant release rate over time, suggests that the hydrophobic EC matrix provided a controlled-release system that maintained steady drug release. Furthermore, the Korsmeyer-Peppas model revealed an n value of 0.64, indicating an anomalous transport mechanism, where drug release was controlled by a combination of diffusion and matrix relaxation, with minor swelling of the hydrophobic polymer [56,57]. For the E_10_H_5_/CPM formulation, the release kinetics were best described by the Korsmeyer-Peppas model (R^2^ = 0.9959, n = 0.40). The n value of 0.40 in the Korsmeyer-Peppas model indicated that the drug release followed a Fickian diffusion mechanism, where CPM molecules diffused through the hydrated polymer matrix in response to a concentration gradient [58,59]. This was further supported by its excellent fit to the Higuchi model (R^2^ = 0.9877), which is characteristic of diffusion-controlled release systems. The enhanced drug release observed in E_10_H_5_/CPM can thus be attributed to the improved diffusion pathways created by the hydrophilic and swellable HPMC within the matrix.

Overall, the release profiles of E_10_/CPM and E_10_H_5_/CPM demonstrated the impact of polymer composition on the drug release mechanism. E_10_/CPM exhibited a controlled release with a predominantly diffusion- and relaxation-based mechanism, while E_10_H_5_/CPM facilitated a more efficient release through Fickian diffusion, largely due to the hydrophilic and swelling properties of HPMC. These findings highlight the importance of selecting appropriate polymers to modulate the release kinetics and enhance the therapeutic efficacy of floating tablet formulations.

## 4. Conclusions

This study highlights the potential of the sugar templating method as an effective and sustainable approach for fabricating highly porous floating tablets using cellulose derivatives. The optimized E_10_H_5_/CPM formulation exhibited superior performance in terms of porosity (94.4%), immediate buoyancy, and extended floating time (>24 h), making it a promising candidate for GRDDS. The mechanical strength evaluation indicated that the incorporation of 5% *w*/*w* HPMC enhanced structural integrity, as evidenced by a higher tensile strength (0.156 MPa) compared to EC-only formulations. Moreover, the addition of HPMC significantly improved the drug release profile, enabling 79.01% of the loaded CPM to be released over 72 h. Micro-CT imaging further confirmed the homogeneity of the pore structure, which played a crucial role in maintaining buoyancy and controlled drug release of the developed formulation. While the formulation successfully achieved prolonged floating and sustained release, a slight reduction in drug loading capacity (6.40 mg in E_10_H_5_/CPM vs. 8.72 mg in E_10_/CPM) was observed. These findings underscore the need for further optimization in polymer composition or alternative solvent systems (e.g., aqueous-organic solvent mixtures) to maximize drug entrapment efficiency. While sugar templating offers a simpler and more cost-effective approach compared to other manufacturing techniques, its scalability requires further investigation. To achieve industrial feasibility, key parameters such as the uniformity of sugar templates, the efficiency of sugar leaching, and the robustness of the polymer matrix must be optimized. Additionally, developing a continuous manufacturing process or integrating this method with existing pharmaceutical production lines could enhance its applicability. A further study on direct comparison with other novel manufacturing techniques (e.g., 3D printing and hot-melt extrusion) is also essential to evaluate the advantages and limitations of sugar templating in terms of drug release control, mechanical properties, and manufacturing feasibility. Additionally, future research should also focus on exploring the incorporation of poorly water-soluble drugs to further reduce drug loss and improve drug loading efficiency, as well as conducting *in vivo* studies to validate the clinical applicability of these formulations and assess their pharmacokinetic performance.

## Figures and Tables

**Table 1:** Composition of cellulose derivative-based floating tablets.

Formulation Code	EC (% *w*/*w*)	HPMC E5 (% *w*/*w*)	Acetone (qs. to %)
E_7.5_	7.5	-	100
E_10_	10.0	-	100
E_12.5_	12.5	-	100
E_15_	15.0	-	100
E_7.5_H_5_	7.5	5.0	100
E_10_H_5_	10.0	5.0	100
E_12.5_H_5_	12.5	5.0	100
E_15_H_5_	15.0	5.0	100
E_7.5_H_7.5_	7.5	7.5	100
E_10_H_7.5_	10.0	7.5	100
E_12.5_H_7.5_	12.5	7.5	100
E_15_H_7.5_	15.0	7.5	100

**Table 2:** Physical properties of drug-loaded floating tablets.

Formulation Code	Weight (mg ± SD)	Diameter (mm ± SD)	Thickness (mm ± SD)	Density (g/cm^3^ ± SD)
E_10_/CPM	39.93 ± 0.25 ^a^	12.23 ± 0.04 ^a^	4.30 ± 0.16 ^a^	0.079 ± 0.003 ^a^
E_10_H_5_/CPM	50.40 ± 3.69 ^b^	12.43 ± 0.08 ^b^	4.39 ± 0.14 ^a^	0.095 ± 0.010 ^b^

For each test, average values with the same letter are not significantly different. Thus, average values with the different letters, e.g., ‘a’ or ‘b’, are statistically different (*p* < 0.05).

**Table 3:** Mechanical strength, floating behavior, and CPM loading content of drug-loaded floating tablets.

Formulation Code	Radial Tensile Strength (MPa ± SD)	CPM Loading Content (mg ± SD)	Floating Lag Time (min)	Total Floating Time (h)
E_10_/CPM	0.143 ± 0.045 ^a^	8.72 ± 0.53 ^a^	0	>24
E_10_H_5_/CPM	0.156 ± 0.004 ^b^	6.40 ± 0.22 ^b^	0	>24

For each test, average values with the same letter are not significantly different. Thus, average values with the different letters, e.g., ‘a’ or ‘b’, are statistically different (*p* < 0.05).

**Table 4:** Release kinetic data for drug-loaded floating tablets fitted to various mathematical models.

Kinetic Model	Parameters	Formulation Code	
		E_10_/CPM	E_10_H_5_/CPM
Zero-order	$R^{2}$	0.9901	0.9451
	$k_{0}\;({min}^{-1})$	1.43	3.73
First-order	$R^{2}$	0.9439	0.8614
	$k_{1}\;({min}^{-1})$	0.25	0.17
Higuchi matrix	$R^{2}$	0.9695	0.9877
	$k_{H}\;({min}^{1/2})$	4.52	12.15
Korsmeyer–Peppas	$R^{2}$	0.9900	0.9959
	$k_{K}\;({min}^{-n})$	3.19	16.60
	n	0.64	0.40

## Data Availability

Dataset available on request from the authors.

## Supplementary Materials

The following supporting information can be downloaded at: https://www.mdpi.com/article/10.3390/polym17040485/s1, Figure S1: Macroscopic images of (a) E_10_/CPM and (b) E_10_H_5_/CPM.
