# Supplementary material for: Fabrication of Cellulose Derivatives-Based Highly Porous Floating Tablets for Gastroretentive Drug Delivery via Sugar Templating Method
Source: Polymers (Basel). 2025 Feb 12;17(4):485. doi: 10.3390/polym17040485 (PMC11859971; doi:10.3390/polym17040485)
Supplement: Supplementary file 1 [file polymers-17-00485-s001.zip › polymers-3448142-supplementary.pdf]

Supplementary Materials: Fabrication of Cellulose Derivatives-Based Highly Porous Floating Tablets for Gastroretentive Drug Delivery via Sugar Templating Method

Pattaraporn Panraksa [1], Tanpong Chaiwarit [1], Baramee Chanabodeechalermrung [1], Patnarin Worajittiphon [2] and Pensak Jantrawut [1,*]

[1] Department of Pharmaceutical Sciences, Faculty of Pharmacy, Chiang Mai University, Chiang Mai 50200, Thailand; pattaraporn.pan@cmu.ac.th (P.P.), tanpong.ch@cmu.ac.th (T.C.), barameechana@gmail.com (B.C.)
[2] Department of Chemistry, Faculty of Science, Chiang Mai University, Chiang Mai 50200, Thailand; patnarin.w@cmu.ac.th
* Correspondence: pensak.j@cmu.ac.th; Tel.: +66-539-443-09

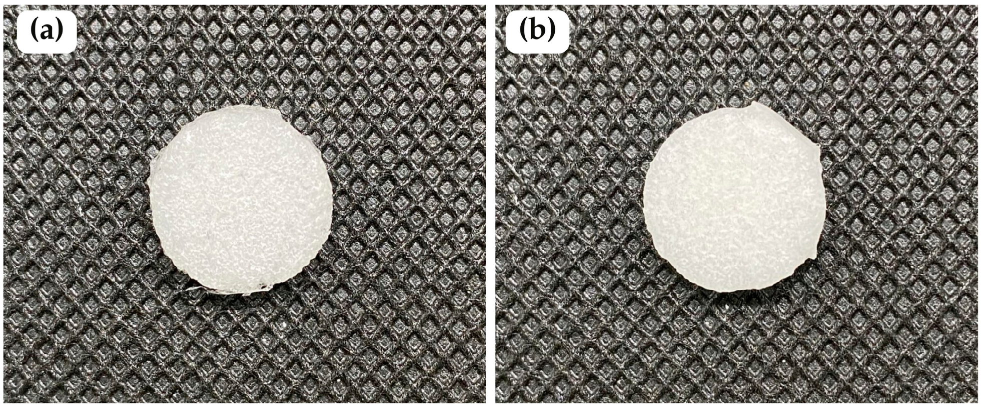

Figure S1. Macroscopic images of (a) E_{10}/CPM and (b) $E_{10}H_5$/CPM.
